# Renal Manifestations of Drug Reaction with Eosinophilia and Systemic Symptoms (DRESS) Syndrome: A Systematic Review of 71 Cases

**DOI:** 10.3390/jcm12144576

**Published:** 2023-07-10

**Authors:** Marilia Dagnon da Silva, Sidney Marcel Domingues, Stevan Oluic, Milan Radovanovic, Pratyusha Kodela, Terri Nordin, Margaret R. Paulson, Bojan Joksimović, Omobolanle Adetimehin, Devender Singh, Cristian Madrid, Milena Cardozo, Marko Baralic, Igor Dumic

**Affiliations:** 1 Municipal University of São Caetano do Sul—USCS Bela Vista, São Paulo 09521-160, Brazil; mariliadagnon@gmail.com (M.D.d.S.); sidusp@gmail.com (S.M.D.); 2 Department of Internal Medicine, Loyola University Medical Center, Maywood, IL 60402, USA; stevan.oluic@luhs.org; 3 Mayo Clinic College of Medicine and Science, Rochester, MN 55905, USA; radovanovic.milan@mayo.edu (M.R.); nordin.terri@mayo.edu (T.N.); paulson.margaret@mayo.edu (M.R.P.); adetimehin.omobolanle@mayo.edu (O.A.); singh.devender@mayo.edu (D.S.); madrid.cristian@mayo.edu (C.M.); cardozo.milena@mayo.edu (M.C.); 4 Department of Hospital Medicine, Mayo Clinic Health System, Eau Claire, WI 54703, USA; 5 Mayo Clinic Health System, Eau Claire, WI 54703, USA; pratyushakodela@gmail.com; 6 Department of Family Medicine, Mayo Clinic Health System, Eau Claire, WI 54703, USA; 7 Faculty of Medicine Foca, University of East Sarajevo, 73300 Foca, The Republic of Srpska, Bosnia and Herzegovina; bojannjoksimovic@gmail.com; 8 Department of Nephrology, Mayo Clinic Health System, Eau Claire, WI 54703, USA; 9 Department of Nephrology, University Clinical Center of Serbia, 11000 Belgrade, Serbia; baralicmarko@yahoo.com; 10 School of Medicine, University of Belgrade, 11000 Belgrade, Serbia

**Keywords:** acute kidney injury, drug hypersensitivity syndrome, DRESS syndrome, drug reaction, eosinophilia, renal, kidney, hematuria, proteinuria, glomerulonephritis, DIHS

## Abstract

Unlike other adverse drug reactions, visceral organ involvement is a prominent feature of drug reaction with eosinophilia and systemic symptoms (DRESS) syndrome and correlates with mortality. The aim of this study was to systematically review cases published in PubMed-indexed, peer-reviewed journals in which patients had renal injury during the episode of DRESS syndrome (DS). We found 71 cases, of which 67 were adults and 56% were males. Female sex was associated with higher mortality. Chronic kidney disease (CKD) was present in 14% of patients who developed acute kidney injury (AKI) during DS. In 21% of cases, the kidneys were the only visceral organ involved, while 54% of patients had both liver and kidney involvement. Eosinophilia was absent in 24% of patients. The most common classes of medication associated with renal injury in DS were antibiotics in 34%, xanthine oxidase inhibitors in 15%, and anticonvulsants in 11%. Among antibiotics, vancomycin was the most common culprit in 68% of patients. AKI was the most common renal manifestation reported in 96% of cases, while isolated proteinuria or hematuria was present in only 4% of cases. In cases with AKI, 88% had isolated increase in creatinine and decrease in glomerular filtration (GFR), 27% had AKI concomitantly with proteinuria, 18% had oliguria, and 13% had concomitant AKI with hematuria. Anuria was the rarest manifestation, occurring in only 4% of patients with DS. Temporary renal replacement therapy was needed in 30% of cases, and all but one patient fully recovered renal function. Mortality of DS in this cohort was 13%, which is higher than previously reported. Medication class, latency period, or pre-existing CKD were not found to be associated with higher mortality. More research, particularly prospective studies, is needed to better recognize the risks associated with renal injury in patients with DS. The development of disease-specific biomarkers would also be useful so DS with renal involvement can be easier distinguished from other eosinophilic diseases that might affect the kidney.

## 1. Introduction

The term “Drug ***Rash*** with Eosinophilia and Systematic Symptoms” was first used in the mid-twentieth century to refer to a set of signs and symptoms resulting from a severe hypersensitivity reaction to a particular drug or its metabolite [1]. After realizing that skin rash was not a mandatory feature for diagnosis, the nomenclature changed, and the syndrome was renamed “Drug ***Reaction*** with Eosinophilia and Systematic Symptoms” (DRESS) syndrome, also known as “Drug-induced Hypersensitivity Syndrome” (DIHS) [1].

The condition usually manifests 2 to 6 weeks after initiation of the culprit medication and may occur up to 3 months after exposure [2]. While many medications can cause DRESS syndrome (DS), anticonvulsants, antimicrobials, and xanthine oxidase inhibitors (e.g., allopurinol) are the most common culprits [2,3,4].

DS typically manifests with a skin rash, facial edema, leukocytosis with eosinophilia and/or presence of atypical lymphocytes, fever, lymphadenopathy, and multiple organ dysfunction in severe cases [2,3]. The diagnosis of DS is based on clinical and laboratory criteria. It is particularly important to exclude other alternative diagnoses such as infection, neoplasm, and autoimmune disease [5]. The European Registry of Severe Cutaneous Adverse Reactions (RegiSCAR) is the most used diagnostic tool for DS worldwide. Based on the RegiSCAR score, DS can be classified as: no case (score < 2), possible (score 2–3), probable (score 4–5), and definitive DS (score ≥ 6) [5,6].

DS is a rare but potentially fatal disease. Its incidence ranges from 1:1000 to 1:10,000 people, with a mortality rate as high as 10% [2,7]. Visceral involvement in DS is directly associated with increased mortality [8]. The most affected visceral organ is the liver while involvement of other internal organs (kidneys, lungs, pancreas, colon, and heart) is less common [8,9,10]. After the liver, the next most frequently involved organ is the kidney [2,4]. Renal injury in DS is defined as abnormal findings of serum urea and creatinine, a decrease in creatinine clearance and glomerular filtration rate (GFR), as well as the presence of proteinuria, hematuria, and eosinophiluria [4].

Due to the rarity of this syndrome, conducting prospective studies is not practical. Current available literature includes single-institution retrospective studies, case reports, case series, and systematic or scoping reviews. This systematic review, therefore, aims to synthesize data on the renal manifestations of DS by reviewing case reports and case series published on this topic. 

## 2. Materials and Methods

### 2.1. Search Strategy, Definitions, and Selection Criteria

**Figure 1:**
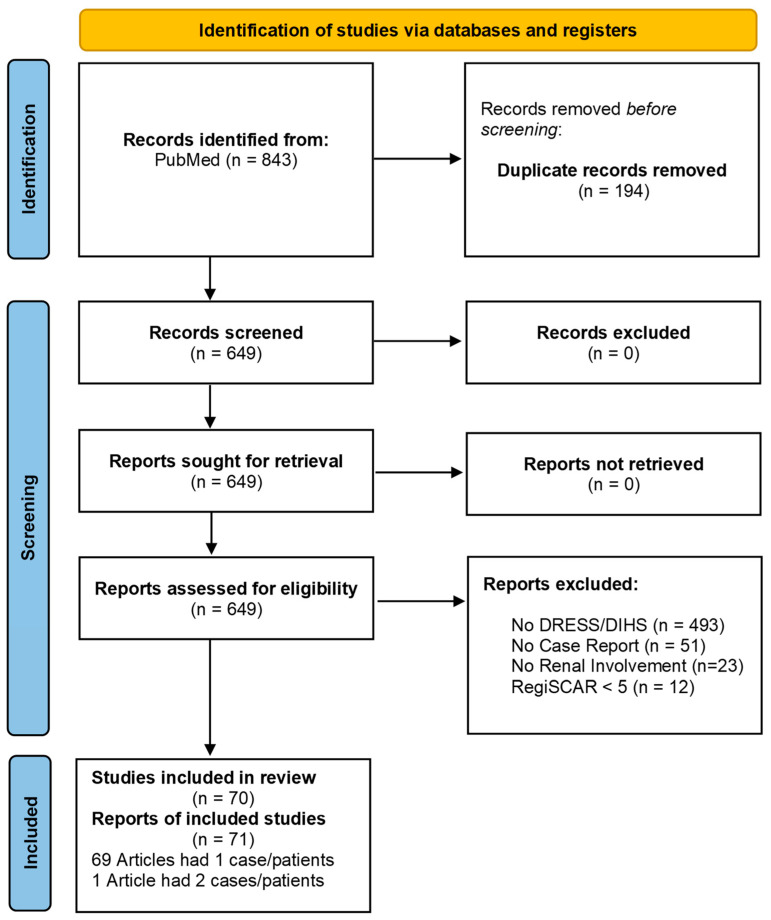
Flowchart demonstrating a selection process according to PRISMA guidelines.

This systematic review was carried out according to Preferred Reporting Items for Systematic Reviews and Meta-Analyses (PRISMA) guidelines (Figure 1). The included references dated from January 2002 to December 2022 and were extracted from the Medline database (National Library of Medicine, Bethesda, MD, USA) through the PubMed search engine during the month of December 2022. The following keywords (a combination of *MeSH* and *non-MeSH* terms) were used: “DRESS AND ACUTE KIDNEY INJURY”, “DRESS AND RENAL”, “DRESS AND KIDNEY”, “DRESS AND HEMATURIA”, “DRESS AND PROTEINURIA”, “DRESS AND GLOMERULONEPHRITIS”, “DIHS AND ACUTE KIDNEY INJURY”, “DIHS AND RENAL”, “DIHS AND KIDNEY”, “DIHS AND HEMATURIA”, “DIHS AND PROTEINURIA”, and “DIHS AND GLOMERULONEPHRITIS”. We used two search filters: articles in English; publications from the last twenty years (since 2002). We analyzed cases of DS with renal involvement in which patients met the criteria for probable and definitive DS according to the RegiSCAR score.

The included cases demonstrated clear kidney involvement as a part of DS. The renal involvement was defined as abnormalities in blood or urine markers of kidney function: increase in serum creatinine (≥0.3 mg/dL within 48 h or at least 1.5 times the baseline from the last seven days), decreased glomerular filtration rate (GFR < 60 mL/min), oliguria (a urine volume < 0.5 mL/kg/h in an interval of six hours or 0.3 mL/kg/h in 24 h), hematuria (≥3 red cells per high-power field in at least one urinalysis) [11,12], and/or proteinuria (presence of >150 mg of protein in the 24 h urine collection) [13]. Patients were considered to have chronic kidney disease (CKD) if the authors reported CKD as a diagnosis in the past medical history section of the case report.

For a patient with pre-existing CKD, we included only cases that demonstrated clear worsening of the renal function during DS and where authors also considered kidney function abnormalities to be due to DS.

The latency period was defined as the time in days elapsed between the administration of the culprit drug and the appearance of the first symptoms. We included both pediatric and adult patients. 

### 2.2. Data Collection and Statistical Analysis

Case reports were manually screened by the first author (MDS), and the senior author (ID) provided clarification where necessary before the final selection. The Rayyan software was also used as a tool, which consists of a web application developed by QCRI (Qatar Computing Research Institute) which was responsible for the process of screening articles and removing duplicates.

We entered all selected articles into a spreadsheet and extracted the following data: demographic data, comorbidities, immunosuppression, social history, latency period, culprit medication, number of visceral organs affected, degree of eosinophilia, RegiSCAR score, imaging tests, serology for cytomegalovirus (CMV), herpes virus 6 and 7 (HHV-6 and HHV-7), Epstein–Barr virus (EBV), treatment, length of hospitalization, sequelae, and outcome (death). As for the articles that did not have all the information that would later be tabulated, it was placed as “not reported” in the data sheet.

We used methods of descriptive and analytical statistics. In descriptive statistics, we used measures of central tendency and measures of variability, namely: arithmetic mean with standard deviation and relative numbers for categorical variables. The nonparametric chi-square or Fisher test and parametric *t*-test were used for independent samples or nonparametric alternatives. Mann–Whitney test was used to compare differences between groups for the univariate analysis of risk factors associated with mortality. Finally, a binary logistic regression model was utilized for the multivariate analysis to assess the possible association of risk factors with mortality. All statistical analyses were performed using IBM SPSS Statistics Software version 24.0 for Windows (IBM Corp., Armonk, NY, USA). All *p*-values lower than 0.05 were considered statistically significant.

## 3. Results

### 3.1. Literature Search

The initial search of the Medline database over the span of 20 years yielded 843 records, of which 194 were duplicates. We screened and assessed the titles and abstracts of all 649 non-duplicate records, excluding 579 articles irrelevant to the topic. A total of 70 articles yielded 71 cases that fulfilled the inclusion criteria for analysis [5,14,15,16,17,18,19,20,21,22,23,24,25,26,27,28,29,30,31,32,33,34,35,36,37,38,39,40,41,42,43,44,45,46,47,48,49,50,51,52,53,54,55,56,57,58,59,60,61,62,63,64,65,66,67,68,69,70,71,72,73,74,75,76,77,78,79,80,81,82]. See Figure 1 for the flow chart of detailed article selection and final studies included.

### 3.2. Demographics and Comorbidities

**Figure 2:**
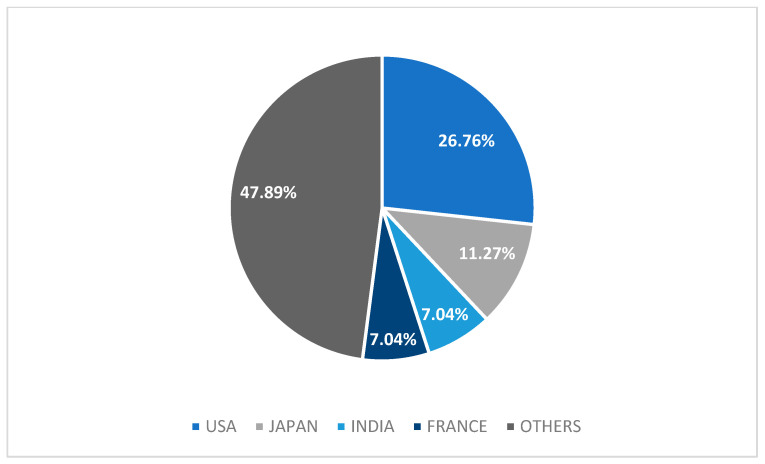
Graph of origin countries of case reports. Others: China, Korea, Tunisia, Spain, Turkey, Belgium, Australia, Switzerland, United Kingdom, Germany, Egypt, Canada, Brazil, Taiwan, Ukraine, Bulgaria, Ecuador, Croatia, Portugal, Netherlands, and Peru.

We analyzed 67 adult (94.4%) and 4 (5.6%) pediatric cases. Most cases were male (56%) (Table 1). In univariate analysis, female sex was associated with higher mortality (*p* = 0.034), but this significance was lost in multivariate analysis (*p* > 0.05) (Table 2). Out of 71 cases, only 10 (14%) cases reported pre-existing CKD (Table 2). Most cases originated from the United States (26.8%), followed by Japan (11.3%), India (7%), and France (7%) (Figure 2).

### 3.3. Visceral Organ Involvement

**Figure 3:**
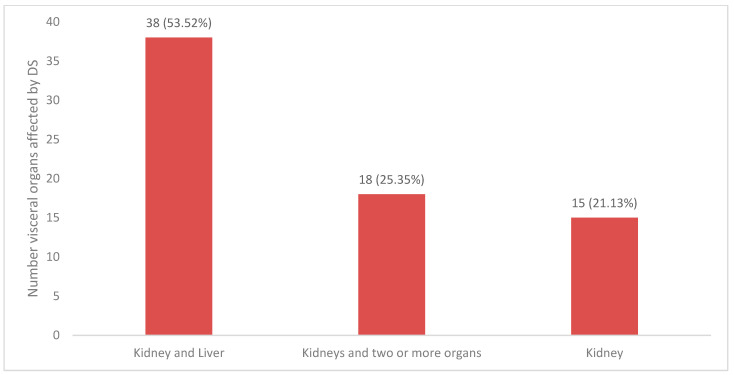
Graph of visceral organs affected by DRESS. Others: kidney/lungs/liver, kidney/lungs/heart, kidney/thyroid/heart/liver, kidney/liver/heart, kidney/liver/spleen, kidney/intestine, kidney/liver/pancreas/lungs, kidney/thyroid, kidney/liver/brain, kidney/liver/eye, kidney/liver/heart/lung.

Kidneys were the sole organ involved in 15 cases (21.1%). Combined kidney and liver involvement was the most common combination and was encountered in 38 cases (53.5%), while more than three visceral organs (including kidneys) were seen in the remaining 25.3% of cases (Figure 3). In multivariate analysis, the number of internal organs involved did not correlate with mortality (*p* > 0.05) (Table 2).

### 3.4. Causative Drugs and Latency

**Figure 4:**
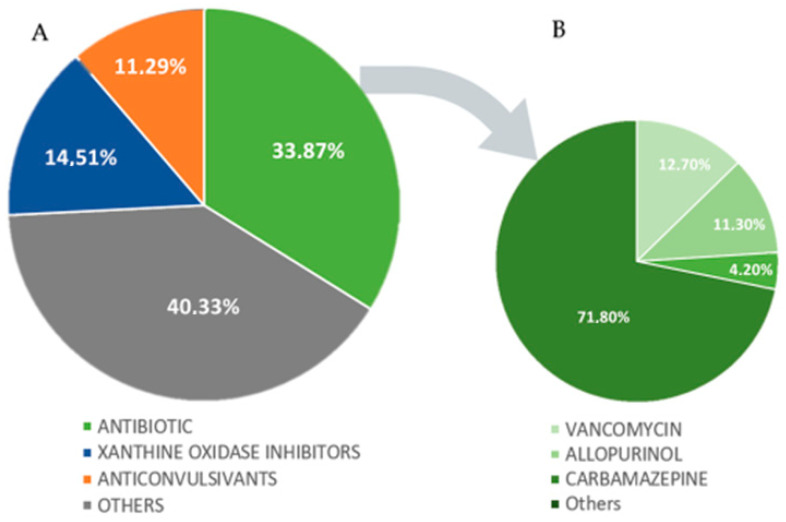
(**A**,**B**). Graphs of the distribution of causative agents by medication class and by medication (**A**): Others: tyrosine kinase inhibitors, immunomodulatory, non-steroidal anti-inflammatory drugs (NSAIDs), diuretic, antirheumatic, proton-pump inhibitors, antithyroid, antitubercular, oxazolidinones, titanium bioprosthesis, analgesics, monoalkylamines, antipsychotics, alkylating agents, acid oxidation inhibitors, 3-hidroxi-3-methyl-glutaril-CoA reductase inhibitors, anticoagulant, indanones, and dpp-4 inhibitors. (**B**): Others: amoxicillin, nitrofurantoin, trimethoprim-sulfamethoxazole, ciprofloxacin, vemurafenib, phenytoin, strontium ranelate, sitagliptin, diaphenylsulfone, clopidogrel, ethambutol, quetiapine, febuxostat, sorafenib, fluindione, titanium bioprosthesis, furosemide, omeprazole, ibuprofen, propylthiouracil, lamotrigine, rosuvastatin, leflunomide, sodium valproate, lenalidomide, spironolactone, chlorambucil, sulphasalazine, cefepime, trimetazidine, minocycline, cyanamide, clindamycin, zonisamide, acetaminophen, linezolid, and meropenem.

A solo agent was identified as the culprit of DS in 62 cases (87.3%), with more than one agent as the probable cause of DS in 9 cases (12.7%). Antibiotics were the most common class of medications identified, followed by xanthine oxidase inhibitors and anticonvulsants (Figure 4A). The most common medications found to cause DS were vancomycin (12.7%), allopurinol (11.3%), and carbamazepine (4.2%), as shown in Figure 4B.

### 3.5. Eosinophilia 

Eosinophilia of varying levels was present in 52 patients (76%) ranging from 635 to 23,200 cells/mcL (average 3815 ± 4789 cells/mcL) and was absent in 16 patients (24%). The level of eosinophilia did not correlate with mortality. 

### 3.6. Viral Reactivation

Most cases (43, 60.6%) reported information regarding viral reactivation. Cytomegalovirus (CMV) was positive in 7 patients (16%), Epstein–Barr virus (EBV) in 9 (21%), and human herpes virus 6 (HHV-6) in 10 cases (23%), while in 17 cases (40%), there was no viral reactivation to any of these viruses.

### 3.7. Renal Manifestations

**Figure 5:**
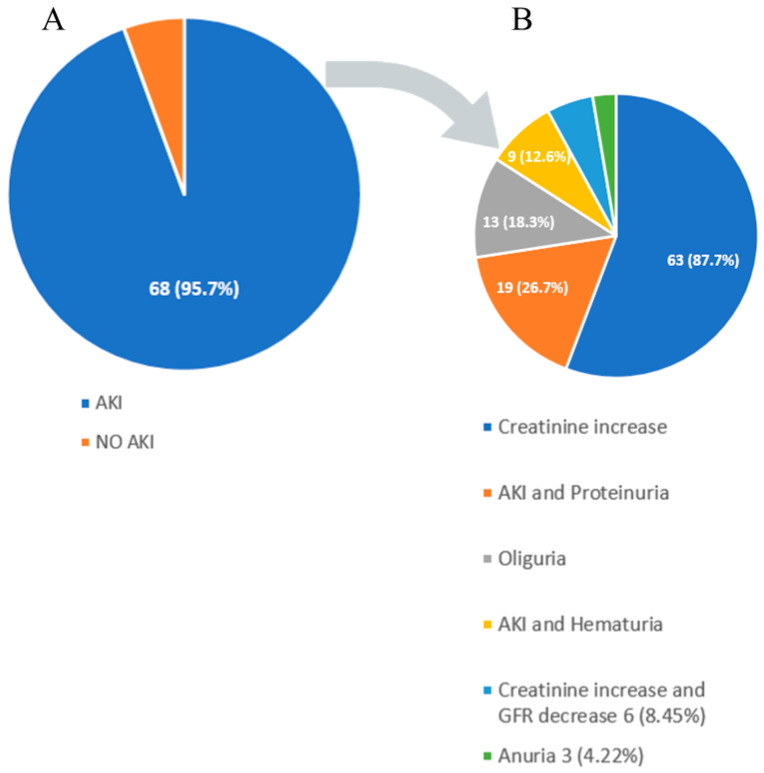
(**A**,**B**) Graph of various kidney manifestations of DRESS syndrome. AKI: acute kidney injury.

Renal manifestations in DS are diverse [4]. AKI was observed in 68 patients (96%), while 3 patients (4%) had isolated proteinuria and hematuria without AKI. Oliguria and anuria were reported in 13 (18%) and 4 (6%) cases, respectively. Patients with AKI, in addition to alteration in creatinine and GFR, also exhibited hematuria in 9 (13%) cases, proteinuria in 19 (27%), while AKI, proteinuria, and hematuria together were present in 6 (8%) patients (Figure 5).

### 3.8. Renal Imaging

Kidney imaging was performed in only 22 patients (31%). Of the patients who underwent imaging tests, the most common modality was renal ultrasonography (US) in 21 cases (95.4%). Abdominal computed tomography (CT) was performed in 11 patients (50%), while magnetic resonance imaging (MRI) was performed in only 1 patient (4.5%) (Table 3). 

Ultrasonographic changes such as a wider prominent pyramid of the kidney, increased renal size, and dilation of calyces, hepatosplenomegaly, and ascites have been reported. In cases that underwent CT, the changes seen included inguinal lymphadenopathy in the aortic and iliac chains, splenomegaly, and ascites. The lone MRI performed showed no abnormalities. 

### 3.9. Renal Biopsy

Renal biopsy was performed in 18 cases (25.3%) (Table 3), with the most common finding being acute interstitial nephritis (14 cases, 77.8%). Less common findings on biopsy were granulomatous lesions, tubular necrosis, interstitial edema, tubular atrophy, and interstitial fibrosis. Abundant cellular infiltrates in reported cases consisted of eosinophils, lymphocytes, plasma cells, neutrophils, and macrophages. Vasculitis was identified in two patients.

### 3.10. Treatment, Sequelae, and Outcome

Hospitalization for DS with renal manifestation was required in 100% of cases and lasted from 4 to 157 days (average of 32.9 ± 33.6 days). Of all 71 cases reviewed, 69 (97.2%) reported the medication used in the treatment of the patient with DS. Monotherapy with steroids was the most common (62.3% of cases), followed by steroids along with another immunosuppressive medication (26.1% of cases). In 11.6% of cases, steroids were not used.

Among all patients, 21 (29.6%) underwent renal replacement therapy (RRT) (Table 2), and 8 did not report the duration of RRT. Among the cases that reported the duration of RRT, the average duration was 13.8 ± 22.8 days. Of these, 12 required therapy for a short period, and 1 reported the need for long-life hemodialysis [24]. Finally, only one case (1.4%) reported that the patient affected by DS progressed to CKD [24].

In the analysis, we found no statistically significant association between sequelae and the causative drug (*p* > 0.05). Sequelae noted after the resolution of DS included two cases of type 1 diabetes mellitus [20,41], two cases of type 2 diabetes mellitus [33,78], one case of autoimmune thyroiditis [41], and one case with both herpes zoster and vitiligo [20].

Of the 71 total cases, 69 reported the patient outcomes: 87% of patients survived DS, while 13% died (Table 1). Table 2 shows that the only observed difference in mortality between groups was sex, with significantly more women who died when compared to men (*p* = 0.034). Of all female cases (n = 31), seven (9.9%) died, while of all male cases (n = 40), only two (2.8%) died. Furthermore, we noted that no pediatric patient died. Between groups divided by mortality, the other differences in other possible risk factors listed in Table 3 were not observed. Table 4 also shows no other significant risk factors for mortality in this scoping review.

## 4. Discussion

To the best of our knowledge, this is the first systematic review exploring renal manifestations during DS. The specific type of kidney injury seen in DS varies. Some studies reported the rate of renal injury as low as 5.2% [83], while others reported as high as 65% [84]. In most other retrospective studies, this number was somewhere between these two extremes at around 15–35% [85,86,87,88,89,90,91] (Table 5).

### 4.1. Demographics and Comorbidities

In this review, the univariate analysis revealed that prognosis tends to be worse in females and with higher mortality. This finding supports what Toniato et al. found, in that the female sex increases the chance of severe DS [86]. This finding is interesting since some prior studies on visceral manifestations of DS did not report similar findings. For example, a recent review on cardiac manifestations of DS did not report sex having any influence on mortality [10]. This same study showed that age above 65 years was correlated with higher mortality [10], while we did not find such a correlation in this study examining renal manifestations of DS. 

Regarding comorbidities, some studies concluded that age over 68 years and the presence of a high number of comorbidities are associated with more severe DS [86]. Furthermore, a study of 60 cases showed that the presence of chronic kidney disease may increase the risk of renal involvement [85,90]. Interestingly, another retrospective study that analyzed 34 cases did not find any patients with renal involvement [93].

Recent reviews on pediatric DS described that presenting features, including renal manifestations, and outcomes are similar to the adult population [89,96,98]. While many studies reported slight female predominance [84,85,86], we find slightly more men in this review. One of the possible explanations is that patients on allopurinol (a common causative agent in DS with renal manifestation) is prescribed for gout, which is more common in men than women.

### 4.2. Visceral Organ Involvement

In our study, the majority of patients who had renal involvement simultaneously had hepatic involvement, which is not surprising as the liver is the most affected visceral organ in DS, followed by the kidneys, as shown by retrospective studies [92,94,95]. In a review that addressed only pediatric patients, the lungs were the most affected organ after the liver, with renal involvement being the third most observed [96]. This pattern was also observed in an observational study that analyzed both adult and pediatric patients [83]. Recent studies showed that patients with hepatic involvement in DS were more likely to have associated renal involvement [99,100]. In our multivariate analysis, we did not observe a clear difference in mortality in patients with several visceral organs involved, which is consistent with the DS study with cardiac involvement [10].

### 4.3. Causative Drugs and Latency

This review identified antibiotics as the drug class most frequently causing DS, with vancomycin being the most common. Similar findings of antibiotics as the most common class of medication associated with DS were observed in other retrospective studies [98,100], a case series [101], and in recent literature reviews [8,99]. Allopurinol, which was associated with a higher risk of renal involvement in some studies [85,90], is the second most common causative agent of DS in this review. Interestingly, anticonvulsants appeared as the third most common in our review, and in many previous studies, it was identified as the main causative class [89,95,96,101,102,103]. It remains to be answered in future studies if certain medication classes have the predisposition to cause particular visceral manifestations of DS.

The period between the use of medication and the onset of symptoms (latency) in our review ranged from 0.5 to 60 days, which is somewhat shorter than what was reported in previous studies, which went as high as 105 days [83,87,91,94]. The latency period in DS may vary according to the medications that cause the syndrome, especially with antibiotics for which the latency period might be particularly short [104]. Like other studies [100,102], we found no statistically significant correlation between the latency period and the causative drug, although Sandhu et al. showed in their observational study that a shorter latency period was seen with the use of antibiotics [97,104]. Allopurinol, in turn, was associated with a long latency period [85], as well as carbamazepine [83,87]. The mean latency period of anticonvulsants and non-anticonvulsant drugs was statistically significant in some studies [84,94].

In our review, we did not observe an association between the latency period and the prognosis; however, in a review on DS with heart involvement, a short latency (<15 days) was related to higher mortality [10], and in another on DS with involvement of the lungs, a latency period equal to or less than 30 days was associated with the development of ARDS [8].

### 4.4. Pathophysiology

The pathophysiology of DS is very complex and not yet fully understood. We know that DS is dose-independent and has an idiosyncratic reaction. Patients’ genetic makeup, particularities about specific drug metabolism and its metabolites, viral reactivation, and complex interplay between these factors all contribute to DS development [3,99,105,106,107,108,109,110,111].

### 4.5. Eosinophilia and Differential Diagnosis

The revised 2016 World Health Organization (WHO) classification of eosinophilic disorders divides these conditions into primary and secondary. While primary causes are purely hematologic (clonal) in nature, secondary (reactive) causes include numerous other conditions, such as infections, non-myeloid malignancies, autoimmune diseases, allergic and atopic conditions, drug reactions, collagen-vascular diseases, and metabolic conditions such as adrenal insufficiency. By this definition, DS belongs to the reactive, secondary eosinophilic disorder.

The International Cooperative Working Group on Eosinophil Disorders (ICOG-EO) divides these conditions into three categories based on the number of circulating cells. The condition is classified as peripheral blood eosinophilia (PBE) if the number of eosinophils is between 500 and 1500 per microliter of blood (μL), hypereosinophilia (HE) if eosinophils are >1500 (μL) on two examinations (4 weeks apart) and/or tissue HE, and hypereosinophilic syndrome (HES) if eosinophils are >500 (μL) along with the presence of organ damage and/or dysfunction attributable to tissue HE after the exclusion of other reasons for major organ damage [112,113].

When associated with kidney damage, these conditions are mainly reactive (secondary) in nature and can be divided into three main categories:

(I) Hypersensitivity reactions (AIN, DS);
(II) Autoimmune diseases (EGPA, anti-GBM disease);
(III) Other (Kimura’s disease, TINU syndrome, IgG4-RD).

AIN—acute interstitial nephritis, EGPA—eosinophilic granulomatosis with polyangiitis, anti-GBM—anti-glomerular basement membrane disease, TINU syndrome—tubulointerstitial nephritis and uveitis syndrome, IgG4-RD—immunoglobulin G4-related disease

DS with renal involvement is important to distinguish from other disorders with similar presentations of eosinophilia and AKI. For example, acute interstitial nephritis (AIN) is a condition characterized by an inflammatory infiltrate in the kidney interstitium and can be caused by drugs, autoimmune conditions, infections, or idiopathic conditions. Drug-induced acute interstitial nephritis (DI-AIN) is responsible for up to 85% of cases of AIN [114] and is mainly caused by non-steroidal anti-inflammatory drugs (NSAIDs), proton-pump inhibitors, and antibiotics. DI-AIN can be hard to distinguish from DS as it presents similarly with the triad of rash, fever, and eosinophiluria. In cases of DS, another internal organ is usually involved (liver, kidneys, lungs, etc.). In DI-AIN, typical features of DS, such as lymphadenopathy and facial swelling, are absent. In such cases, it is important to calculate the RegiSCAR score and take appropriate history regarding the latency period, as DS usually has a longer latency period than other severe cutaneous adverse reactions (SCAR). 

Eosinophilic granulomatosis with polyangiitis (EGPA) is an autoimmune condition characterized by systemic necrotizing vasculitis of small and medium-sized blood vessels as well as eosinophil-rich tissue infiltrates and granulomatous lesions. The prevalence of anti-neutrophil cytoplasmic antibody (ANCA) positivity in patients with EGPA is about 40%, and renal involvement is found to be closely related to ANCA positivity. ANCA is usually absent in patients with DS. On biopsy, necrotizing pauci-immune glomerulonephritis is the most common renal presentation, found in 88% of ANCA-positive EGPA cases with renal involvement [115], while in DS, interstitial nephritis is the most common histopathological finding. 

Most studies showed highly elevated eosinophil counts in patients with EGPA based on the American College of Rheumatology or Lanham criteria with a median around 8000 (μL), with some groups reporting milder but still significant elevation of eosinophils [116]. Skin findings are completely different in patients with EGPA and DS. The rash occurs in 40–50% of patients with EGPA and mainly presents as palpable purpura of the legs and scalp but also can present as vesicular lesions, urticarial lesions, or necrotic ulcers [117], which differs from the DS rash, which usually presents as a pruritic maculopapular rash or diffuse erythematous eruption. Other specific findings present in patients with EGPA (history of asthma, paranasal sinus abnormality, and mono- or polyneuropathy) are not present in patients with DRESS. 

Anti-glomerular basement membrane (anti-GBM) is another type of vasculitis that needs to be considered in patients with concomitant renal and pulmonary involvement with eosinophilia. This small-vessel vasculitis, caused by an autoimmune reaction against type IV collagen, usually manifests as glomerular necrosis and crescent formation, followed by alveolar hemorrhage due to pulmonary capillary involvement. Making the exact diagnosis sometimes requires renal biopsy accompanied by immunochemistry testing. 

Kimura’s disease is a benign and rare inflammatory disease presenting as painless masses affecting subcutaneous areas of the head and neck (parotid glands, salivary glands, lymph nodes) that can be accompanied by nephrotic syndrome. It mainly affects Asian males and is characterized by elevated immunoglobulin E (IgE) levels and peripheral blood eosinophilia [118]. Space-occupying lesions seen in Kimura’s disease are not characteristic of DS. Additionally, DS does not have racial predominance, and there is no specific skin rash associated with Kimura’s disease. 

Tubulointerstitial nephritis and uveitis (TINU syndrome) is a rare oculorenal inflammatory condition affecting mostly younger patients with an estimated prevalence from <0.1% to 2% in patients of all ages and up to 2.3% in kids [119]. Besides bilateral anterior uveitis (redness, pain, and photophobia), patients may present with peripheral blood eosinophilia, fever, rash, and kidney damage. Typically, patients with DRESS have an absence of ocular symptoms whereas ocular symptoms are predominant and mandatory for diagnosis of TINU syndrome. 

Immunoglobulin G4-related disease (IgG4-RD) is an immune-mediated condition that can affect any organ and cause irreversible fibrosis. It manifests with lymphoplasmacytic infiltration of organs with a high percentage of IgG4 + plasma cells and mild to moderate tissue eosinophilia. It is reported that around 20–40% of patients with this disease have peripheral blood eosinophilia and 51–86% have tissue eosinophilia [120]. The possible multiorgan involvement is similar to both IgG4-RD and DS, however storiform fibrosis of affected organs is not specific to DRESS. Skin manifestations may be present in patients with IgG4-RD and, if present, are mainly nodules (40.4%), papules (36.5%), or plaques (32.7%). This is in contrast to the maculopapular rash, the most common type of rash in patients with DS [121].

Eosinophilia can also be seen in patients on renal replacement therapy (RRT), such as hemodialysis or peritoneal dialysis-associated eosinophilia. Kidney transplant recipients should also be considered separately for possible acute allograft rejection if they present with eosinophilia, and finally, patients with renal cell carcinoma can demonstrate peripheral blood eosinophilia (PBE) [122].

### 4.6. Viral Reactivation

Viral reactivation is often demonstrated in DS cases [3,106,108,123,124]. In some patients with DS who develop thrombosis, onset has been associated with CMV reactivation [125]. Since CMV has the propensity to infect endothelial cells, this finding has been attributed to endothelial dysfunction due to CMV reactivation [125,126]. Reactivation of HHV-6 and the occurrence of autoimmune processes of the thyroid gland have been observed, which is unsurprising since abundant presence of the viral agent has been observed in Hashimoto’s disease [127].

In our analysis, HHV-6 was the virus in which reactivation was most commonly reported. In six out of seven patients in a study that analyzed the causes of multiorgan failure, HHV-6 reactivation was a poor prognostic factor in high-risk patients [91]. We found no statistically significant relationship between viral reactivation and prognosis in our analysis.

### 4.7. Clinical Manifestations of Renal Involvement

The diagnosis of DS is based on clinical criteria and laboratory abnormalities. The symptomatology of DS can sometimes be nonspecific; however, some frequent findings include fever, lymphadenopathy, and, although not mandatory, rash, which may be present in up to 100% of cases [92,128]. Hematological alterations frequently seen in laboratory analyses include leukocytosis with eosinophilia (>0.4 × 10^9^ L) and/or the presence of atypical lymphocytes [129]. The degree of eosinophilia in our patient cohort did not show a statistically significant difference in terms of mortality, findings that are similar to prior studies on visceral manifestations of DS [8,10,100]. We also did not find a significant correlation between eosinophil count and the drug causing DS, contrasting with observations from a retrospective study in which allopurinol was associated with high eosinophilia [85].

Renal involvement can range from mild AKI to severe renal failure requiring renal replacement therapy (RRT) [83,85,88,90,91]. In our study, almost all patients met the criteria for AKI. The increase in the serum creatinine value was the most common finding in our review, just as in several studies that evaluated DS cases in general [83,84,85,88,90,91,92,97,98,100].

Proteinuria with or without AKI is a marker of renal injury in DS, which is also true for hematuria. Given these findings, it is of utmost importance not to rely only on creatinine and GFR values but also to obtain urinalysis in all patients where DS is suspected or diagnosed. The exact percentage of various types of renal involvement is illustrated in Figure 5 and Table 2.

### 4.8. Renal Imaging

Renal imaging in patients with DS who have renal involvement is nonspecific. Intraabdominal lymphadenopathy is the most common finding (Figure 3), while changes in renal parenchyma are nonspecific and cannot be used for diagnostic purposes. We suspect that renal imaging was likely performed in many of the reported cases but not always reported by authors if the imaging revealed no significant findings. Based on the findings from this study, we do not recommend routine renal imaging in patients with DS unless uncertainty remains regarding the etiology of AKI, and obstruction needs to be ruled out. 

### 4.9. Renal Biopsy Findings

A renal biopsy is a powerful diagnostic tool used to diagnose many diseases that affect the kidneys, such as DI-AIN. The pathohistological analysis provides important data regarding the cause of renal injury [114,130,131]. In addition to drug-induced nephritis, as seen in DS, other causes of nephritis include infectious, autoimmune, paraneoplastic, and idiopathic [132,133].

Interstitial edema, glomerular sclerosis, and tubular atrophy may also be seen, while vessels and glomeruli may remain intact despite the presence of interstitial edema [133]. In our review, vasculitis was present in two cases [14,82]. In the first, the findings consisted of necrotizing vasculitis, especially in the intralobular arteries [82]. In the second, there was a granulomatous interstitial pattern associated with vasculitis [14]. In DI-AIN, the most common findings consist of interstitial infiltrates in which plasma cells, lymphocytes, monocytes, neutrophils, and histiocytes are present; however, eosinophils are also commonly found [114,131]. Interestingly, the findings may vary according to the medication that caused the injury, so in DI-AIN secondary to beta-lactam antibiotics or NSAIDs, monocytes are the cells most commonly found in the infiltrates, while eosinophils are rarely found [131]. It was demonstrated in a retrospective study that patients with DI-AIN who progressed to CKD had more interstitial infiltration of inflammatory cells when compared to those who did not progress [134].

The presence of a significant number of eosinophils, although not mandatory, points to drug-induced causes such as DS, while the predominance of neutrophils suggests nephritis of bacterial origin, and the presence of plasmocytes can often be observed in viral cases [131]. The presence of granuloma might be related to medications, although it can also be observed in autoimmune and infectious etiologies [131]. Tubulitis, which can also be seen in renal transplant rejection, is often found with biopsies performed in patients with DI-AIN, and in these cases, lymphocytes are often present in contact with the tubular epithelium [131,135]. Luminal ectasia, apoptotic remnants, and cytoplasmic simplification can also be observed, and the condition may become chronic, evolving into fibrosis and atrophy [131].

There are no pathognomonic signs on immunofluorescence and electron microscopy in patients with DS, but the loss of parts of the podocyte is a common finding in cases of injury by anti-inflammatories [131,133].

Analysis revealed the presence of interstitial nephritis in most cases of DS reviewed seen with granulomatous lesions, tubular atrophy, interstitial fibrosis, infiltration of mononuclear cells, eosinophils, lymphocytes, macrophages, and plasmocytes [14,15,17,20,22,23,31,32,36,39,41,43,50,56,63,64,69,77]. Tubular necrosis and vasculitis in some renal vessels were also observed [82]. In one case, immunofluorescence revealed a granular pattern of at least IgA, IgG, IgM, and C3 in the mesangial region [41].

### 4.10. Treatment and Outcomes

Withdrawal of the offending medication is of utmost importance and should be the first step in the management of any patient with DS. The pharmacotherapy of choice consists of systemic corticosteroids, mainly prednisone and methylprednisolone [90,91]. The use of these medications as the first line of treatment has been advocated for decades, being a widespread therapy in the treatment of immune-related diseases [136].

Recent studies describe the possibility of alternative agents as first-line therapies for DS rather than waiting until cases are refractory to steroids [137,138]. Thus, treatment options such as cyclosporine, mycophenolate, intravenous immunoglobulins (IVIG), mepolizumab, and plasmapheresis are gaining more attention [137,138].

Cyclosporine is a calcineurin inhibitor that prevents the proliferation of T cells and has emerged as a promising agent, even as a first-line therapy in DS [136,139]. Although it is classically reserved for refractory cases, it has shown positive results regarding early introduction in DS cases, and its short use seems to be safer and more beneficial than the prolonged use of steroids in these patients [136,140]. In addition to being a viable option for patients intolerant to glucocorticoids [138], cyclosporine poses less concern in regard to viral reactivation that has been previously attributed to steroids. Additionally, patients treated with cyclosporine might have a favorable clinical evolution, reduction in hospitalization and treatment time, and lower likelihood of experiencing subsequent relapse [136,138,139,140,141].

Plasma exchange therapy (TPE) promotes the decrease in existing systemic cytokines in the context of inflammation and has sometimes been used in the treatment of refractory or life and life-threatening DS [142]. TPE has shown a reduction in morbidity and mortality of patients with steroid-resistant conditions [142,143]. A severe DS case in a pediatric patient with involvement of at least six visceral organs, respiratory failure, and cardiac arrest reported the use of TPE [143]. Another pediatric patient with DS developed hemodynamic and organ function deterioration even after the use of systemic steroids and subsequently was treated with TPE [142].

Martinez et al. reported a case of a young adult patient diagnosed with DS that progressed to anuric acute renal failure, undergoing renal replacement therapy [144]. In this case, the patient showed improvement in the clinical picture only after a session of leukapheresis and granulopheresis. Leukapheresis has been studied and used in the treatment of hypereosinophilia, although it is classically used to treat hyperleukocytosis [144].

The use of mepolizumab for the treatment of DS was first reported in 2017 by Ange et al. and since then its use has been replicated in several case reports [145,146]. This drug is an anti-interleukin-5 monoclonal antibody that blocks the pathway of this interleukin so that T cells are unable to recruit eosinophils, which would result in the control of the clinical condition [147]. This medication has been used successfully in other eosinophilic diseases, such as severe eosinophilic asthma, hypereosinophilic syndrome, and even eosinophilic granulomatosis with polyangiitis [148]. Mepolizumab is another option that can be considered in steroid-resistant cases of DS [146,147,149,150,151]. Damsky et al. reported the use of tofacitinib in two patients with significant cardiac involvement in whom the use of corticosteroids was insufficient [152]. Both had a favorable clinical evolution with the normalization of organic functions, a decrease in the eosinophil count, and lower IL-5 levels [152]. In another study, this medication allowed the control of DS in a patient who had previously failed therapies with systemic steroids, intravenous immunoglobulin, cyclosporine, and mycophenolate [153].

In this review, most patients received systemic corticosteroids as therapy, although we found no statistically significant difference regarding prognosis or duration of treatment between patients who used systemic steroids and those who did not use steroids as the first-line treatment option. Similar findings were observed in patients with cardiac involvement, as reported by Radovanovic and co-authors [10], and in patients with liver involvement [100,154]. Patients with DS who develop renal injury often require RRT until renal function normalizes. In this scoping review, we found that almost 30% of patients required RRT, yet patients usually recover after a variable amount of time and only rarely progress to end-stage renal dialysis requiring life-long RRT [90]. In our analysis, only one case reviewed had life-long hemodialysis evolving and chronic kidney disease [24]. In this review, we found no statistically significant correlation between this therapy and overall outcomes.

We found a higher mortality rate in DS with renal involvement than in DS overall, 13.04% compared to around 10% [1,155]. Additionally, studies that analyzed DS with involvement of other visceral organs noted higher mortality rates than baseline DS at 15.7%with involvement of the entire gastrointestinal tract excluding the liver [102] and 20% with pulmonary involvement [91]. Studies that analyzed hepatic involvement indicated rates between 11% and 25% [100,154,156]. The highest mortality rate reported was 45.2% in patients with cardiac involvement [10]. In a review of the literature on DS in pediatric patients [96], the mortality rate was lower (3%) than when compared to the adult population in DS; however, we found no difference in mortality between the two groups in our study.

### 4.11. Sequelae

DS is associated with an increased risk of development of various complications that might occur concomitantly or after acute DS is resolved. Among these, autoimmune polyendocrine syndrome is very common [125,157].

Sequelae frequently observed in DS are related to autoimmunity; however, the reason for this development is not yet fully known [41,125]. Type I diabetes mellitus, autoimmune hemolytic anemia, and disseminated intravascular coagulation tend to occur early, while rheumatoid arthritis, vitiligo, and alopecia areata usually occur later. Myocarditis, pneumonitis, and autoimmune thyroiditis can occur at any time [128]. Myocarditis and cardiac complications in 30% of cases can develop after typical manifestations of DS have resolved [10].

Kano et al. observed that 13.79% of patients with DS had some type of autoimmune or infectious sequelae during the follow-up period [125]. In the study by Sasidharanpillai et al., which included 40 patients with DS, the frequency of new diseases after the resolution of the syndrome was 10%, and 1 patient developed CKD [158]. Hashimoto’s disease, painful thyroiditis, and Graves’ disease develop more commonly in younger patients [125,159,160,161,162,163].

Lupus erythematosus with severe lupus nephritis developed four years after the onset of DS in a patient who was treated exclusively with IVIG [93].

The appearance of herpes zoster and cryptococcal pneumonia was also observed, mainly during DS; however, both seem to be related to immune inflammatory reconstruction [125].

Finally, it is important to note that the development of comorbidities such as type 2 diabetes mellitus can also be secondary to the prolonged use of corticosteroids in patients with DS. Our analysis did not find any statistically significant association between sequelae occurrence and the causative drug. 

Optimal follow-up of the patient after complete recovery from DS is extremely important as recurrence can occur at any time after the resolution of the condition, ranging from months to years [129,164,165].

## 5. Conclusions

Manifestations of renal injury in patients with DS include creatinine elevation, decrease in GFR, oliguria, anuria, proteinuria, and hematuria. It is important to note that not all patients present with AKI and creatinine elevation, and some might have isolated proteinuria or hematuria. Hence, we recommend routine urinalysis in patients with DS to evaluate for renal involvement. The most common culprit is vancomycin, and about 30% of patients require RRT. The majority of patients recover renal function completely after RRT, and only one case reported progression to CKD and ESRD. The female sex is associated with a worse prognosis, and mortality is 13%, which is higher than previously reported at 10%. Renal imaging is non-specific and we do not recommend routine renal imaging in patients with DS and renal involvement. If there is diagnostic uncertanty renal biopsy should be carried out to rule out other differential diagnosis.

Limitations of the study come from the methodology of this type of review. While we tried to select only high-quality publications, including cases from peer-reviewed journals indexed in PubMed, some cases may be omitted by this strategy. Publication bias (not all cases of DRESS syndrome get reported) which is inherent to this type of review, is probably present to some degree, as well as the fact that not all information of interest was reported in all cases. Other limitations is a relatively small sample size and the inclusion of cases published only in English and Portuguese.

## Figures and Tables

**Table 1:** Illustrates demographic characteristics, comorbidities, viral reactivation, and prognosis of the cases analyzed in this scoping review. Since not all case reports documented all information, we have created column reported to illustrate how many cases reported the variable of interest.

Demographic Characteristics			
Sex			
	Female	31 (31/71, 43.66%)	
	Male	40 (40/71, 56.34%)	
Age (Years)			
	≤18	4 (4/71, 5.63%)	
	19–64	41 (41/71, 57.75%)	
	≥65	26 (26/71, 36.62%)	
Comorbidities			
	Reported	Positive	Negative
CKD	14 (14/71, 19.72%)	10 (10/14, 71.43%)	4 (4/14, 28.57%)
HTN	27 (27/71, 38.02%)	21 (21/27, 77.78%)	6 (6/27, 22.22%)
Immunosuppression	5 (5/71, 7.04%)	3 (3/5, 60%)	2 (2/5, 40%)
Viral Reactivation			
	Reported	Positive	Negative
CMV	43 (43/71, 60.56%)	7 (7/43, 16.28%)	36 (36/43, 83.72%)
HHV-6	43 (43/71, 60.56%)	10 (10/43, 23.26%)	33 (33/43, 76.74%)
EBV	43 (43/71, 60.56%)	9 (9/43, 20.93%)	34 (34/43, 79.07%)
Prognoses			
	Reported	Positive	Negative
Sequelae	7 (7/71, 9.86%)	6 (6/7, 85.71%)	1 (1/7, 14.29%)
	Reported	Discharged	Death
Outcome	69 (69/71, 97.18%)	60 (60/69, 86.96%)	9 (9/69, 13.04%)

CKD = chronic kidney disease; CMV = cytomegalovirus; HHV-6 = human herpesvirus 6; EBV = Epstein–Barr virus.

**Table 2:** Illustrates the risk factors and their association with mortality. Mann–Whitney, chi-square test, Fisher test, and *t*-test were used to compare differences between groups for the univariate analysis of risk factors associated with mortality.

Variables	Alive	Died	Univariate
	N (%) or Mean ± SD	N (%) or Mean ± SD	*p*-Value
Female sex	24 (40.0)	7 (77.8)	0.034 *
Age, years	51.8 ± 20.3	62.5 ± 14.7	0.075 **
Chronic kidney disease present	8 (72.7)	2 (66.7)	0.837 *
Latency, days	22.0 ± 15.2	24.7 ± 17.7	0.643 **
Organs involved, number	2.0 ± 0.8	2.2 ± 0.4	0.277 **
Renal manifestation			
Oliguria	10 (76.9)	2 (100.0)	0.749 *
Anuria	2 (15.4)	0 (0.0)	
Oligoanuria	1 (7.7)	0 (0.0)	
Creatinine peak, mg/dL	4.7 ± 3.6	4.2 ± 2.6	0.693 **
Creatinine high values	49 (92.5)	8 (88.9)	0.717 *
Proteinuria present	19 (86.4)	1 (50.0)	0.186 *
Hematuria present	11 (64.7)	0 (0.0)	0.197 *
Eosinophils peak	4088.9 ± 5062.5	2712.2 ± 3512.9	0.327 **
Eosinophilia present	46 (85.2)	7 (77.8)	0.573 *
RegiSCAR score	6.2 ± 0.9	6.7 ± 1.8	0.290 **
Probable	13 (21.7)	3 (33.3)	0.439 *
Definite	47 (78.3)	6 (66.7)	
Complications (hemodialysis)	16 (84.2)	3 (75.0)	0.659 *
Hemodialysis, days	23.7 ± 33.3	21.0 ± 33.8	0.902 **
Improvement in renal function, days	66.92 ± 132.2	9.00 ± 4.3	0.255 **
CMV, positive	7 (18.9)	0 (0.0)	0.339 *
HHV7, positive	0 (0.0)	0 (0.0)	1.000 *
EBV, positive	8 (21.6)	0 (0.0)	0.300 *
Therapy with steroids			
Steroids monotherapy	34 (58.6)	8 (88.9)	0.140
Steroids + other therapy	18 (31.0)	0 (0.0)	
other	6 (10.3)	1 (11.1)	
Way of administration of therapy			
Per oral	8 (28.6)	1 (33.3)	0.414
i.v.	10 (35.7)	2 (66.7)	
Combination	10 (35.7)	0 (0.0)	
Topic steroids	8 (13.3)	0 (0.0)	0.244

CMV—cytomegalovirus; HHV7—human beta herpesvirus 7; EBV—Epstein–Barr virus; * chi-square test or Fisher test; ** *t*-test for independent samples or Mann–Whitney test.

**Table 3:** Description of renal findings registered in the reported cases.

Renal Findings	
Urine output	
Reported	16 (16/71, 22.53%)
Anuria	4 (4/16, 25%)
Oliguria	12 (12/16, 75%)
Proteinuria	
Reported	25 (25/71, 35.21%)
Yes	21 (21/25, 84%)
No	4 (4/25, 16%)
Hematuria	
Reported	18 (18/71, 25.35%)
Yes	11 (11/18, 61.1%)
No	7 (7/18, 38.9%)
Renal biopsy	
Reported	18 (18/71, 25.35%)
Hemodialysis	
Yes	21 (21/71, 29.58%)
Imaging	
Reported	22 (22/71, 30.98%)
Ultrasound	21 (21/22, 95.45%)
CT ABD	11 (11/22, 50%)
MRI Body	1 (1/22, 4.54%)

CT ABD = abdominal computed tomography; MRI Body = whole-body nuclear magnetic resonance.

**Table 4:** The list of possible risk factors and their association with mortality evaluated by binary logistic regression analysis.

Variables		Multivariate
	OR	*p*-Value *
Female sex	3.53	1.000
Age, years	0.07	0.998
Chronic kidney disease present	0.00	0.999
Creatinine high values	0.00	0.998
Proteinuria present	0.21	0.999
Hematuria present	0.92	0.999
Eosinophilia present	0.98	1.000
RegiSCAR score	0.72	0.999
Hemodialysis, days	0.35	0.999
CMV, positive	0.40	0.999

CMV—cytomegalovirus; * chi-square test or Fisher test; OR—odds ratio; * *p*-value for binary logistic regression analyses.

**Table 5:** Articles of particular interest with renal involvement by DRESS.

Study	Country	Most Common Causative Medication	Number of Patients in the Study	% of Patients with Renal Involvement
Ben M’rad M et al., 2009 [88]	France	Antibiotics	24 cases—12 females (50%)	17%
Eshki M et al., 2009 [91]	France	Xanthine oxidase enzyme inhibitor	15 cases—10 females (66.66%)	40%
Um SJ et al., 2010 [87]	Korea	Anticonvulsants	38 cases—20 females (52.6%)	15.8%
Chen YC et al., 2010 [90]	Taiwan	Xanthine oxidase enzyme inhibitor	60 cases—34 females (56.66%)	40%
Walsh S et al., 2013 [92]	U.K.	Anticonvulsants	27 cases—17 females (62.96%)	7.41%
Ushigome Y et al., 2013 [93]	Japan	Anticonvulsants	34 cases—16 females (47.05%)	0.0%
Sasidharanpillai S et al., 2014 [83]	India	Anticonvulsants	26 cases—14 females (53.84%)	7.69%
Sultan SJ et al., 2015 [84]	India	Anticonvulsants	17 cases—9 females (52.9%)	64.7%
Hiransuthikul A et al., 2015 [85]	Thailand	Anticonvulsants	52 cases—37 females (71.2%)	15.4%
Lee JY et al., 2017 [94]	Korea	Anticonvulsants	25 cases—14 females (56%)	28%
Mehrholz D et al., 2017 [95]	Poland	Anticonvulsants	10 cases—7 females (70%)	30%
Metterle L et al., 2020 [89]	USA	Anticonvulsants	130 cases—66 females (50.8%)	15.4%
Kim GY et al., 2020 [96]	USA	Anticonvulsants	148 cases—66 females (44.6%)	16.9%
Toniato A et al., 2021 [86]	Italy	Xanthine oxidase enzyme inhibitor	25 cases—15 females (60%)	37.5%
Sandhu S et al., 2021 [97]	India	Anticonvulsants and anti-infectives	20 cases—8 females (40%)	15%
Bedouelle E et al., 2022 [98]	France	Antibiotics	49 cases—27 females (55.1%)	26.5%

## Data Availability

All data are publically available.
